# Trametinib Inhibits the Growth and Aerobic Glycolysis of Glioma Cells by Targeting the PKM2/c-Myc Axis

**DOI:** 10.3389/fphar.2021.760055

**Published:** 2021-10-21

**Authors:** Mingjun Gao, Jin Yang, Hailong Gong, Yuancai Lin, Jing Liu

**Affiliations:** ^1^ Department of Neurosurgery, Shengjing Hospital of China Medical University, Shenyang, China; ^2^ Liaoning Clinical Medical Research Center in Nervous System Disease, Shenyang, China; ^3^ Key Laboratory of Neuro-oncology in Liaoning Province, Shenyang, China

**Keywords:** aerobic glycolysis, glioma, trametinib, PKM2, c-myc

## Abstract

Gliomas are primary tumors originating from glial progenitor cells. Traditional treatments, including surgery, radiotherapy, and chemotherapy, have many limitations concerning the prognosis of patients with gliomas. Therefore, it is important to find novel drugs to effectively treat gliomas. Trametinib has been shown to inhibit the MAPK pathway and regulate its downstream extracellular-related kinases. It has widely been used in the treatment of BRAF V600E mutant metastatic melanomas. Previous studies found that trametinib can improve the prognosis of patients with melanoma brain metastases. In this study, we investigated the therapeutic effects of trametinib on gliomas *in vivo* and *in vitro*. We found that trametinib can inhibit proliferation, migration, and invasion of glioma cells, while inducing apoptosis of glioma cells. Specifically, trametinib can suppress both the expression of *PKM2* in glioma cells and the transport of PKM2 into the cellular nucleus via suppression of *ERK1/2* expression. However, inhibition of these cellular effects and intracellular glycolysis levels were reversed by overexpressing *PKM2* in glioma cells. We also found inhibition of c-myc with trametinib treatment, but its expression could be increased by overexpressing *PKM2*. Interestingly, when *PKM2* was overexpressed but *c-myc* silenced, we found that the initial inhibition of cellular effects and glycolysis levels by trametinib were once again restored. These inhibitory effects were also confirmed *in vivo*: trametinib inhibited the growth of the transplanted glioma cell tumor, whereas *PKM2* overexpression and *c-myc* silencing restored the inhibition of trametinib on the growth of the transplanted tumor. In conclusion, these experimental results showed that trametinib may inhibit the growth and intracellular glycolysis of glioma cells by targeting the PKM2/c-myc pathway.

## Introduction

Glioma is a highly malignant intracranial primary tumor, accounting for ∼60% of central nervous system tumors. Even if combined with surgery, radiotherapy, and chemotherapy, the 2-years survival rate is only 20%. Therefore, improving the curative effect and survival rate of patients with gliomas are major problems to solve (Zhang et al., 2018). Fortunately, finding a targeted killing therapy for glioblastomas is gradually becoming the core of anti-glioma therapy.

Trametinib is an inhibitor of MEK1/2, which regulates its downstream ERK kinases by inhibiting the MAPK pathway. In May 2013, the FDA approved the use of trametinib as a treatment for metastatic melanomas—and in addition, for lung cancer, renal cancer, thyroid cancer, cholangiocarcinoma, and breast cancer. Trametinib has also proven anti-tumor effects—either alone or in combination with other drugs (Bridgeman et al., 2016; Planchard et al., 2016; Davies et al., 2017; Ikeda et al., 2018; Subbiah et al., 2018). It has been confirmed that oral trametinib can improve the prognosis of patients with melanoma brain metastases, which suggests that an oral safe dose of trametinib can reach effective anti-melanoma therapeutic concentrations through the blood-brain barrier (Davies et al., 2017). Although there are few studies on the molecular mechanism of the anti-tumor effect of trametinib, recent clinical reports have confirmed its sole- or combination-use to produce safe and effective anti-glioma effects (Vander Heiden et al., 2009; Brown et al., 2017). However, no research has reported on its anti-tumor mechanism.

The enhancement of the glycolysis level is a key factor of tumorigenesis. Different from normal tissue cells, tumor cells prioritize aerobic glycolysis to generate energy even under sufficient oxygen conditions—a phenomenon called the “Warburg effect.” Tumor growth can therefore be inhibited by suppressing the glycolysis level (Christofk et al., 2008; Rodríguez-García et al., 2017; Zahra et al., 2020). Furthermore, pyruvate kinase plays an essential role in cell metabolism. Four isomers are present in mammals, namely PKL, PKR, PKM1, and PKM2 (Zahra et al., 2020). Studies have shown that only PKM2 is expressed in cancer cells, and the expression of PKM2 is significantly increased in glioma cells (Altenberg and Greulich, 2004; Christofk et al., 2008). PKM2 not only directly regulates glycolysis, but also regulates other genes related to glycolysis, such as GLUT1 and LDHA (Yang et al., 2012a). Meanwhile, some studies have also shown that PKM2 can regulate proliferation, apoptosis, migration, invasion, and cell cycle of glioma cells (Yang et al., 2012b; Liang et al., 2016).

The MYC gene is a widely studied proto-oncogene, and include C-myc, L-myc, and N-myc. Studies have shown that MYC gene products, especially c-myc, play a key role in the occurrence and development of cancer. C-myc is upregulated in several types of tumor cells, which can regulate various cell functions, including cell growth, proliferation, differentiation, and programmed death (Annibali et al., 2014). Furthermore, there is a regulatory mechanism between PKM2 and c-myc, while c-myc also directly regulates glucose metabolism genes, such as LDHA, GLUT1, and HK2. Interestingly, more studies have shown that c-myc can upregulate the expression of PKM2 by regulating the pre-mRNA shear protein PTB to cut exon 9 of pyruvate kinase gene (David et al., 2010). There is also a positive feedback loop between PKM2 and c-myc, which can seriously reduce the glycolysis level in glioma cells and produce a tumor killing effect. Therefore, the PKM2/c-myc pathway is a potential drug target for glioma treatment.

The purpose of this study is to study the effect of trametinib on the glycolysis level and biological function of glioma cells through the PKM2/c-myc pathway. Trametinib has passed the clinical phase III trial and has been approved by FDA for melanoma treatment. Its safety has been verified to some extent, and studies have shown that, trametinib has a certain therapeutic effect on the recurrence and progression of low-grade gliomas (pLGGs) in children (Manoharan et al., 2020; Selt et al., 2020). This study will hopefully provide new drug targets for the clinical development of glioma drugs. This will be done by clarifying the mechanism of trametinib in killing tumors to provide a new molecular theoretical basis for the application of trametinib in anti-tumor therapy.

## Materials and Methods

### Reagents and Materials

Trametinib and U0126 were purchased from MedChemExpress (Monmouth Junction, NJ, United States). Dimethyl sulfoxide was used to dissolve trametinib powder to an initial concentration of 10 mM and preserved in −80°C. The CCK-8 kit was obtained from Dojindo (Rockville, MD, United States). The Annexin V-FITC/PI apoptosis detection kit and Annexin V-PE/7-ADD apoptosis detection kit were purchased from Nanjing Vazyme Biotech (Nanjing, China). Penicillin and streptomycin were obtained from Gibco (Thermo Fisher Scientific, Waltham, MA, United States). DMEM were purchased from CORNING (Corning Life Sciences, Bedford, MA, Unied States). The fetal bovine serum (FBS) was purchased from TBD (Tianjin, China). Lipofectamine 3,000 and TRIzol™ Reagent were obtained from Life Technologies (Carlsbad, CA, United States). XF96 seahorse assay plate was purchased from Agilent (Santa Clara, CA, United States). The antibodies used were as follows: anti-PKM2 antibody (#15822-AP), anti-c-myc antibody (#67447-1-lg), anti-GLUT1 antibody (#21829-1-AP), anti-LDHA antibody (#19987-1-AP), anti-β-actin antibody (#66009-1-lg), MEK1/2 polyclonal antibody (#11049-1-AP), ERK1/2 polyclonal antibody (#16443-1-AP), and anti-PCNA antibody (10205-2-AP) from Proteintech (Wuhan, China); and anti-Ki67 antibody (ab279653) from Abcam (Cambridge, United Kingdom).

### Cell Culture and Transfection

The cell lines U87 and U251 (human glioma cells) were obtained from Shanghai Gene Chemistry (Shanghai, China). The cells were cytogenetically assessed using STR technology for authentication. Dulbecco’s modified Eagle medium (DMEM) containing 10% serum and 1% penicillin-streptomycin was used to culture the cells, which were placed in an incubator containing 5% carbon dioxide at 37°C. The *PKM2* overexpression and c-myc-shRNA (sh-myc) plasmid was synthesized by GenePharma (Shanghai, China). The target sequence of sh-myc was 5′-GAG​AAT​GTC​AAG​AGG​CGA​ACA-3′, whereas the target sequence of the negative control (sh-vector) was 5′-TTC​TCC​GAA​CGT​GTC​ACG​T-3’. To establish stably transfected U87 and U251, the two cell lines were planted into 24-well plates, respectively. When 80% confluence occurred, cells were transfected with the vectors: PKM2 or sh-myc plasmids. Lipofectamine 3,000 Reagent (Thermo Fisher Scientific) was used for plasmid transfection. We added 0.75 μl lipofectamine 3,000 reagent and 2 μl P3000 reagent to 25 μl opti-MEM Medium according to the instructions, then added 1 μg DNA, and the mixture was added to the 24-well plate, and then incubated at 37°C with 5% CO2 for 24 h, observed the fluorescence of the cells. Suitable cells were screened by G418 and puromycin, while transfection efficiency was measured by qPCR and western blot.

### Cell Viability Assay

The CCK-8 kit was used in accordance with the manufacturer’s instructions to detect trametinib activity against U87 and U251 cells. The two lines were seeded into 96-well plates at a density of 3×10^5^ per well. The treatment time of trametinib on cells was 6, 12, 24, 36, 48, 60, and 72 h, respectively. The CCK8 solution was mixed with DMEM at a ratio of 1:9, and used to replace the solution in the wells. The cells were then incubated at 37°C with 5% CO_2_ for 2 h. Absorbance was measured at 450 nm wavelength to quantify the cell viability.

### Flow Cytometry Analysis of Apoptosis

The cells were first treated with trametinib for separate times in 12-well plates, digested with trypsin, and then resuspended in PBS. To measure apoptosis, the cells were stained by annexin V-FITC and PI, under dark conditions according to the manufacturer’s instructions, and analyzed with a flow cytometer.

### Cell Migration Assays

The HoloMonitor M4 culture system (PHIAB, Lund, Sweden) was used to detect the migration ability of glioma cells treated by trametinib. The trametinib treated cells were put into the system for 24 h, before being analyzed using the Hstudio M4 software.

### Cell Invasion Assays

The invasion ability of the cells was assessed by the transwell method. Cells (5×10^5^) were first seeded into a transwell chamber with Matrigel at the bottom (Corning Life Sciences, Bedford, MA, Unied States). Then, 100 μl serum-free DMEM was used in the transwell chamber, and 600 μl DMEM containing 20% FBS was used in a 24-well plate, while the chamber was incubated for 48 h. The lower layer was fixed with 4% formaldehyde, and then stained with Giemsa staining solution (Leagene Biotechnology, Beijing, China). Five fields were randomly selected under the microscope to count the number of invaded cells.

### Extracellular Acidification Rate Measurement

An XFe96 Analyzer (Agilent) was used to evaluate the level of aerobic glycolysis of cells, according to the manufacturer’s instructions. Subsequently, 50 nM trametinib was treated for 6, 12, 48, and 72 h, respectively. U87 and U251 cells were seeded into XFe96 cell culture plates at a density of 3,000 cells per well. The cells were left to evenly distribute the cells at 25°C for 1 h, and then incubated at 37°C with 5% CO_2_ overnight. The probe card device was hydrated the day before the experiment, before being placed in a 37°C CO_2_-free incubator overnight. On the day of the experiment, pyruvate, glutamine, and glucose were added to the basic culture medium according to the kit instructions at final concentrations of 1 mM, 2 mM, and 10 mM, respectively. The culture solution was then replaced with the test solution, and then placed in a 37°C CO_2_-free incubator for 45 min. The oligomycin, glucose, and 2-deoxyglucose were added following the manufacturer’s instructions. Briefly, the oligomycin, glucose, and 2-deoxyglucose were added to the probe board port, and then the extracellular acidification rate (ECAR) of glioma cells was measured by the XFe96 Extracellular Flux Analyzer (Seahorse Bioscience, Billerica, MA, United States) in real-time.

### Western Blot

Cells were lysed with lysis buffer mixed (1:100) with RIPA buffer (Beyotime, Shanghai, China) and PMSF (Beyotime). The cells broken by the ultrasonic disruptor were then centrifuged at 5,000 *g* at 4 °C for 60 min. The concentration of the supernatant was measured with the BCA kit (Beyotime). The protein sample in the SDS-PAGE gel (Beyotime) was separated with 120 V voltage in SDS-PAGE Electrophoresis Buffer with Tris-Gly. The separated protein was transferred to the PVDF membrane in the Western Transfer Buffer. The PVDF membrane was then placed in the blocking solution for 30 min, and sequentially incubated with primary and secondary antibodies. The western blot visualization was enhanced with an ECL Kit (Beyotime). The β-actin was used to calculate the relative integral density value.

### RNA Isolation and Quantitative Real-Time PCR

TRIzol reagent (Life Technologies Corporation, Carlsbad, CA, United States) was used to lyse the cells and extract RNA from the cells. *PKM2*, *c-myc*, *GLUT1*, *LDHA*, and *β-actin* mRNA was reverse transcribed into cDNA by the HiScriptⅢ RT SuperMix for qPCR kit (Vazyme, Nanjing, China), stained using the ChanQ universal SYBR qPCR Master Mix kit (Vazyme), and detected with the 7,500 Fast RT-PCR System (Applied Biosystems, Waltham, MA, USA).

### Structural Analysis of Trametinib Binding to PKM2

Crystal structures of PKM2 (DB code:3GR4) were downloaded from the RCSB database (https://www.rcsb.org/). The protein structure was determined using AutoDock 4.2 to remove the crystal water and add hydrogens as preparation before docking. The docking site is located at the junction of ATP and FBP. Discovery studio 2.5 software was used to enhance the image after docking.

### Animal Experiment

The animal study was reviewed and approved by the Animal Ethical Committee of Shengjing Hospital of China Medical University (2020PS590K). Trypsin was used to digest the adherent cells cultured in DMEM containing 10% FBS. Live cells were washed and resuspended in PBS. Each 100 ml cell suspension contained 4×10^6^ cells. Cell suspension (100 μl) was injected into the skin with a syringe under the right armpit of BALB/c female nude mice. These mice weighed 14–15 g and were obtained from Beijing Huafukang Biotechnology (China). The mice (*n* = 5 animals per group) were randomized to six treatment groups. They were injected with either untransfected cells, vector-transfected cells, PKM2-overexpressed cells, PKM2+sh-NC cells, or PKM2+sh-myc cells. Trametinib was administered daily at a concentration of 1 mg/kg via oral gavage. The tumor volume was measured using an electronic caliper every 3–4 days and calculated by the formula: length × (width/2) × 0.5.

### Immunohistochemical Staining

Paraffin tissue slices were placed in Xylene solution for 20 min, and then hydrated with anhydrous ethanol. The hydrated slices were placed in a Citrate Antigen Retrieval Solution (Beyotime). Slices were treated by endogenous peroxidase inhibitors for 30 min. ERK1/2 polyclonal antibody (diluted 1:50), PKM2 polyclonal antibody (diluted 1:100), and anti-Ki67 antibody (diluted 1:1,000) were placed on the surface of the slices, which were then placed in a 4°C refrigerator for 12 h. Thereafter, the slices were placed at 25°C for 30 min, adding Biotin labeled lamb anti-mouse/rabbit IgG polymer (MXB Biotechnologies, Fuzhou, China) for 30 min. All slices received streptomycin antibiotic protein-peroxidase (MXB Biotechnologies) for 10 min at 25°C. The Dab HorseraDish peroxidase Color Development Kit (Beyotime) was used to treat the glass sheet, before adding Hematoxylin Staining Solution (Beyotime), and observing the slides under a microscope.

### Statistical Analysis

All quantitative data were expressed as mean ± standard deviation (SD). These data were the results of at least three independent experiments. The images of these data were displayed using GraphPad Prism 8 software (GraphPad Software, San Diego, CA, United States). The data of the two groups were compared by Student’s *t*-tests (unpaired, two-tailed). The one-way analysis of variance (ANOVA) (followed by Tukey’s post-hoc tests) was used to compare the difference between the data and the control. *p* < 0.05 was regarded to be statistically significant: *p < 0.05; **p < 0.01.

## Results

### Trametinib can Induce Apoptosis of Glioma Cells and Inhibit Cell Proliferation, Migration, and Invasion

**FIGURE 1:**
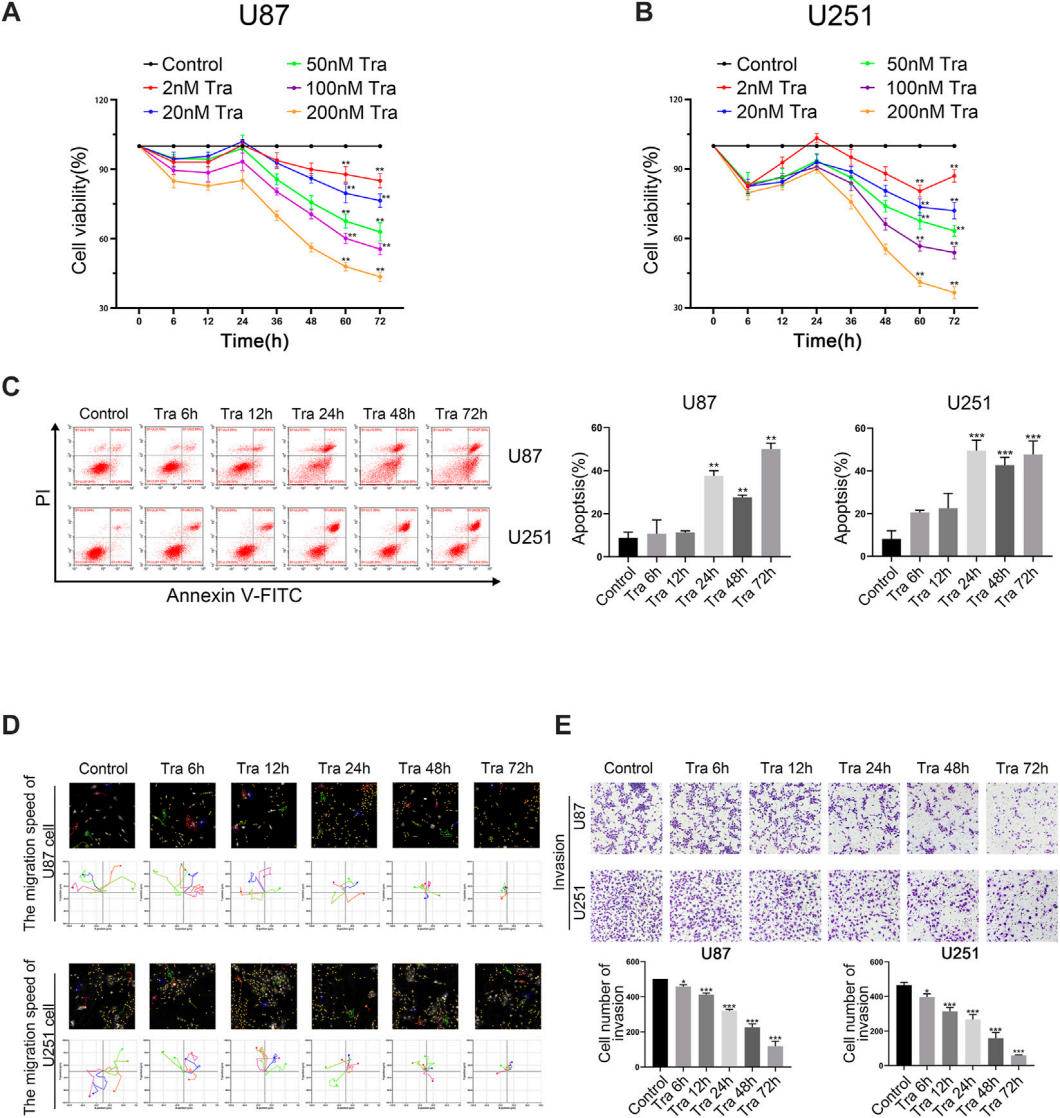
Trametinib induces apoptosis of glioma cells and inhibit cell proliferation, migration, and invasion. (**A**) The toxic effect of trametinib on U87 cells. (**B**) The toxic effect of trametinib on U251. (**C**) U87 and U251 cells were treated with 50 nM trametinib for 0, 6, 12, 24, 48, and 72 h, and the effects of 50 nM trametinib on apoptosis were detected by flow cytometry. (**D**) Quantification of the apoptotic cells. (**E**) The migration ability of U87 and U251 cells was analyzed by Hstudio M4 system after 50 nM trametinib treatment for 0, 6, 12, 24, 48, and 72 h. Scale bars: 100 µm. (**F**) The invasion ability of glioma cells is detected by transwell assay after trametinib treatment for 0, 6, 12, 24, 48, and 72 h. Data are presented as means +SD (*n* = 3); *p < 0.05; **p < 0.01, vs control.

First, we studied the effect of trametinib on the proliferation of U87 and U251 cells. The viability rate of cells was detected by CCK-8. Cells were treated with trametinib at 2, 20, 50, 100, and 200 nM, respectively, for 6, 12, 24, 36, 48, 60, and 72 h, respectively. Trametinib significantly inhibited the growth of U87 (Figure 1A) and U251 cells (Figure 1B). Interestingly, with 24 h treatment, both U87 cells and U251 cells had unknown resistance to drugs of various concentrations. At this time point, the inhibitory effect of drugs on glioma cells did not increase with the increased treatment time as expected, but decreased slightly compared with 12 h of treatment. For treatment times of 48, 60, and 72 h, significant inhibition of the two cell lines occurred by various concentrations of trametinib with significant dose dependence. These results showed that trametinib significantly inhibited the viability of U87 and U251 cells. Considering that the blood-brain barrier will physiologically reduce drug entry from plasma into brain cells, we chose 50 nM trametinib for 48 h treatment to conduct the following experiments.

To further study the role of apoptosis in trametinib toxicity to glioma cells, flow cytometry was used. The apoptosis rate of U87 and U251 cells increased obviously after treatment with 50 nM trametinib for more than 48 h (Figures 1C,D). It can be seen from the figures that the apoptosis of U87 and U251 cells induced by trametinib is mainly caused by late apoptosis. As the time of drug action increases, the cells in the UR quadrant in Figure 1C gradually increase, while the cells in the LR quadrant are almost no change. Therefore, trametinib can induce late apoptosis of glioma cells without the occurrence of early apoptosis. Simultaneously, we used Hstudio M4 software and a transwell experiment to study the effect of trametinib on cell migration and invasion. The migration and invasion ability of glioma cells significantly decreased with increased treatment time when U87 and U251 cells were treated for 6, 12, 24, 48, and 72 h, respectively (Figures 1E,F).

### Trametinib can Inhibit the Aerobic Glycolysis Level in Glioma

**FIGURE 2:**
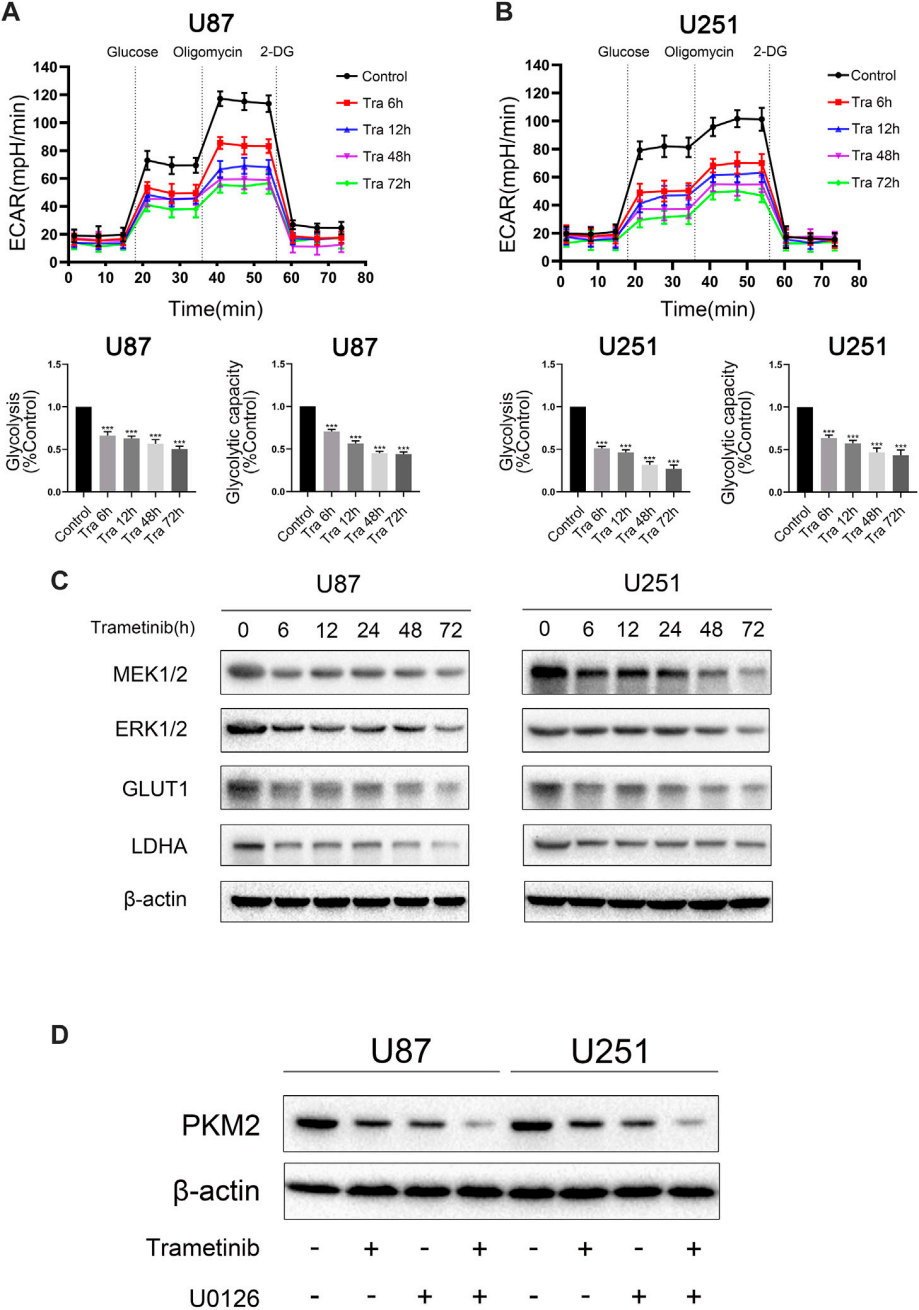
Trametinib inhibits the aerobic glycolysis level in glioma. (**A**) ECAR indicates the level of aerobic glycolysis in U87 cells treated with 50 nM trametinib for 0, 6, 12, 48, and 72 h. The glycolysis and glycolytic capacity were analyzed. (**B**) ECAR indicates the level of aerobic glycolysis in U251 cells treated with 50 nM trametinib for 0, 6, 12, 48, and 72 h. The glycolysis and glycolytic capacity were analyzed. (**C**) Glioma cells were treated with 50 nM trametinib for 0, 6, 12, 24, 48, and 72 h. Total protein was extracted to disclose MEK1/2, ERK1/2, GLUT1 and LDHA levels. (**D**) Effects of trametinib and U0126 on PKM2. Data are presented as means +SD (*n* = 3); *p < 0.05; **p < 0.01, vs control.

Aerobic glycolysis is one of the most significant signs of tumor formation, including gliomas. Therefore, we used the seahorse XF96 to detect the effect of trametinib on the glycolysis level in glioma cells. We chose treatment times of 6, 12, 48, and 72 h in U87 and U251 cells. With the increased treatment time of trametinib on glioma cells, the glycolytic activity and glycolytic reserve of U87 cells (Figure 2A) and U251 cells (Figure 2B) significantly decreased. Accordingly, western blotting and qRT-PCR showed that the glycolytic marker proteins GLUT1 and LDHA significantly decreased in transcription (Supplementary Figure S3A) and translation (Figure 2C). These results indicated that trametinib could inhibit the aerobic glycolysis level of U87 and U251 cells with increased treatment time.

### Trametinib May Inhibit the Expression Activity of PKM2 by Interacting With PKM2

**FIGURE 3:**
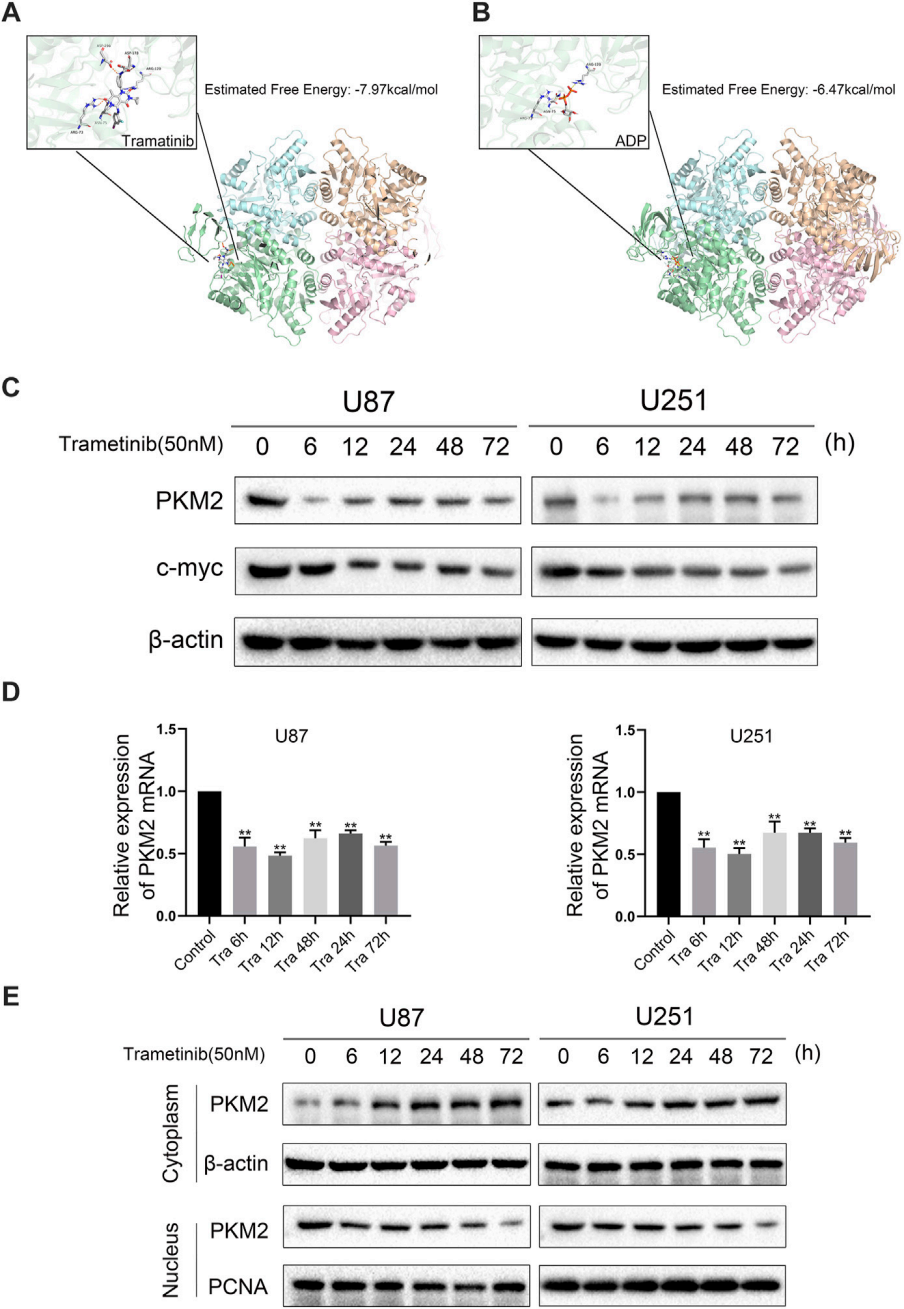
Trametinib may inhibit the expression activity of PKM2 by interacting with PKM2. (**A**) The binding pocket of ADP in the PKM2 tetramer. The predicted free energy of binding for trametinib is −7.97 kcal/mol. (**B**) The binding pocket of ADP in the PKM2 tetramer. The predicted free energy of binding for trametinib is −6.47 kcal/mol. (**C**) U87 and U251 cells were treated with 50 nM trametinib for 0, 6, 12, 24, 48, and 72 h, and the expression levels of *PKM2* and c-myc proteins were detected. (**D**) The mRNA levels of *PKM2* in U87 and U251 cells were detected by RT-qPCR after treatment with 50 nM trametinib for 0, 6, 12, 24, 48, and 72 h (**E**). The location of PKM2 in the glioma cells treated with trametinib by western blotting. Data are presented as means +SD (*n* = 3); *p < 0.05; **p < 0.01, vs control.

To study the mechanism of trametinib-induced glioma cell death, we predicted the possible targets of trametinib with SwissTargetPrediction software and performed a KEGG pathway enrichment analysis. We found that “pathways in cancer” was enriched in the targets of trametinib (Supplementary Figure S1). Furthermore, aerobic glycolysis of tumor cells is an essential sign of tumor formation. As the key rate-limiting enzyme in aerobic glycolysis, PKM2 plays a vital role in the metabolism of cancer cells. According to our research showing trametinib inhibiting glycolysis of glioma cells, we suspect that trametinib interacts with PKM2. We obtained the protein 3D structure of PKM2 (3GR4) from the Protein Data Bank website. Then, using AutoDock 4.2, the energy minimization structures of PKM2 and trametinib were analyzed by molecular docking. We docked trametinib with the FBP binding bag (Supplementary Figure S2A) and ADP binding bag (Figure 3A), respectively, and compared the binding positions of FBP (Supplementary Figure S2B) and ADP (Figure 3B). This revealed that trametinib may have targeted a binding effect on the ADP binding bag of PKM2, thus inhibiting the activity of PKM2. Previous studies have found that ERK1/2 can promote the translocation of PKM2 from the cytoplasm to the nucleus (Yang et al., 2012a). As an inhibitor of MEK1/2, whether trametinib can affect the MAPK pathway by inhibiting the expression of MEK, thereby affecting the function of ERK1/2, reducing the translocation of PKM2 to the nucleus, thus affecting its function remained an open question. Curiously, we found that the expression of MEK1/2 and ERK1/2 in glioma cells after the treatment of trametinib decreased (Figure 2C), and using the MEK/ERK inhibitor U0126 to block ERK1/2 showed that trametinib can further reduce the expression of PKM2 (Figure 2D). To study the effect of trametinib on PKM2 in glioma, the two cell lines were treated with trametinib for 6, 12, 24, 48, and 72 h, respectively. Glioma cells treated with trametinib were detected by either western blotting or qRT-PCR. Because PKM2 can be transferred into the nucleus, to explore the influence of PKM2 on the inhibitory of trametinib, we studied the changes in protein expression of PKM2 in the cytoplasm and nucleus as a result of various trametinib treatment times. The results showed that trametinib had obvious inhibitory effect on PKM2 at both the protein level (Figure 3C) and transcription level (Figure 3D). Trametinib also significantly increased the protein expression of PKM2 in the cytoplasm, but significantly decreased the protein expression of PKM2 in the nucleus (Figure 3E). Interestingly, we also found that trametinib has obvious inhibitory effect on c-myc at both the protein level (Figure 3C) and transcription level (Supplementary Figure S3B). These results indicate that trametinib can not only affect the mRNA expression of PKM2 at the transcription level, but also affects PKM2 at the protein level through direct binding. Trametinib can thus inhibit the transfer of PKM2 protein from the cytoplasm to the nucleus. Simultaneously, trametinib also inhibited the transcription and translation of the protooncogene c-myc.

### Trametinib Inhibits Migration, Invasion, and Glycolysis of Glioma Cells and Induces Apoptosis Through PKM2

**FIGURE 4:**
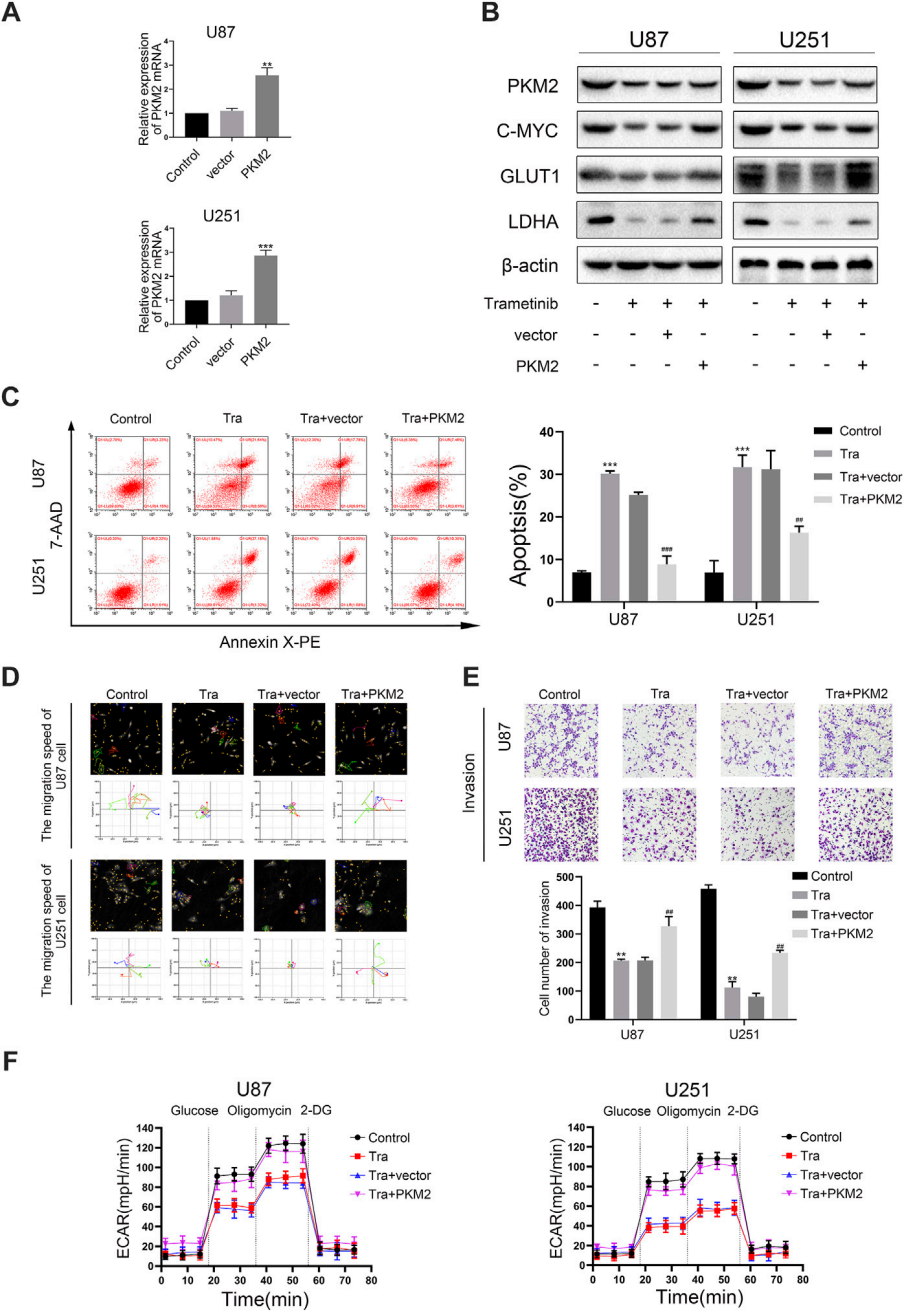
Trametinib inhibits migration, invasion, and glycolysis of glioma cells and induces apoptosis through PKM2. (**A**) *PKM2* mRNA levels was measured in glioma cells transfected with a vector or *PKM2*. (**B**) Expression of c-myc, GLUT1 and LDHA by western blotting upon *PKM2* overexpression after 50 nM trametinib treated for 48 h. (**C**) The effects of 50 nM trametinib treated for 48 h on apoptosis after *PKM2* overexpression was detected by flow cytometry. (**D**) The capacity for migration in U87 and U251 cells on *PKM2* overexpression after 50 nM trametinib treated for 48 h was analyzed by Hstudio M4 software. Scale bars: 100 µm. (**E**) The capacity for invasion in glioma cells on *PKM2* overexpression after 50 nM trametinib treated for 48 h was detected by the transwell method. (**F**) ECAR indicates the level of aerobic glycolysis in glioma cells with *PKM2* overexpression treated with 50 nM trametinib for 48 h. Data are presented as means +SD (*n* = 3); *p < 0.05; **p < 0.01, vs control. ^#^ *p* < 0.05; ^##^ *p* < 0.01, vs Tra + vector group.

Numerous studies have shown that trametinib can significantly suppress the expression of *PKM2* in U87 and U251 cells. To research the influence of PKM2 on migration, invasion, and apoptosis in glioma, we constructed stably expressed *PKM2* overexpressed transfection strains for two cell lines, and evaluated the transfection efficiency by qRT-PCR (Figure 4A). Compared with the vector group, the expression of *PKM2* mRNA in PKM2 overexpression group was significantly higher. Furthermore, *PKM2* expression in the vector group was no different from that in the blank group. Meanwhile, the results of the Hstudio M4 software and transwell experiment showed that the migration and invasion ability of the two cell lines significantly decreased after 50 nM trametinib treatment for 48 h. However, glioma cells overexpressing *PKM2* partially resisted the inhibition of migration and invasion by trametinib (Figures 4D,E). Similarly, seahorse XF96 energy level detection and flow cytometry were used to measure the glycolysis level and apoptosis rate of glioma cells. The glycolysis level of glioma cells significantly decreased, whereas the apoptosis rate significantly increased; the *PKM2* overexpressed glioma cells could also resist the glycolysis level changes and apoptosis changes of trametinib treatment (Figure 4F and Figure 4C). Western blotting (Figure 4B) and qRT-PCR (Supplementary Figure S3C) were used to show that *GLUT1* and *LDHA* expression could be significantly restored in glioma cells with *PKM2* overexpression. These discoveries showed that overexpression of *PKM2* can reverse the influence of trametinib on migration, invasion, glycolysis level, and apoptosis rate in glioma cells, which also indicates that *PKM2* plays an essential role in the mechanism by which trametinib kills glioma.

### Trametinib Regulates the Killing Effect and Glycolysis Level of Glioma Cells Through the PKM2/c-Myc Axis

**FIGURE 5:**
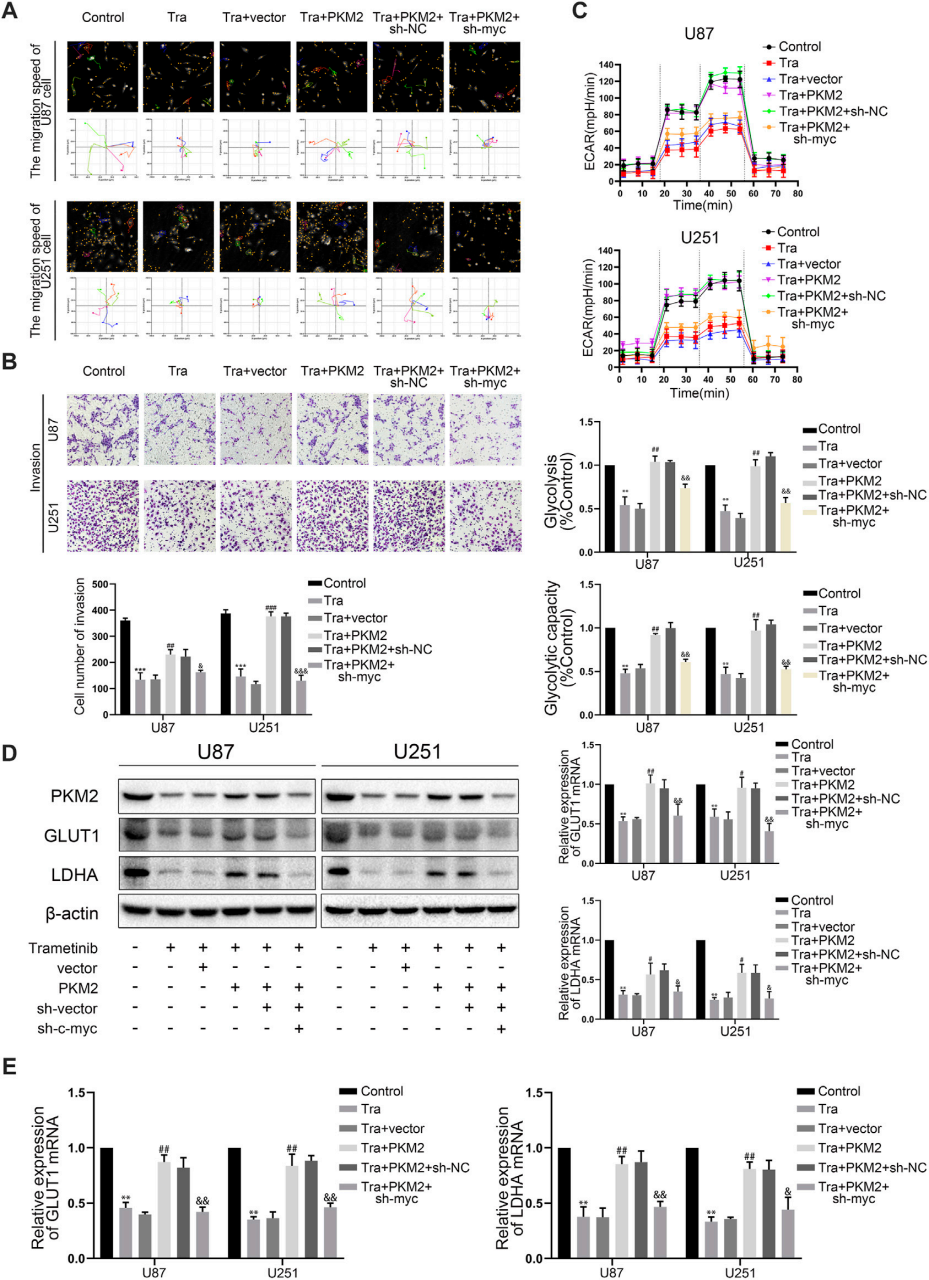
Trametinib regulates the killing effect and glycolysis level of glioma cells through PKM2/C-MYC axis. (**A**) trametinib (50 nM) for 48 h in glioma cells on the capacity for migration was analyzed by Hstudio M4 software. Scale bars: 100 µm. (**B**) trametinib (50 nM) for 48 h in glioma cells on the capacity for invasion was analyzed by transwell. (**C**) trametinib (50 nM) for 48 h in glioma cells on glycolysis was analyzed. (**D**) trametinib (50 nM) for 48 h in glioma cells on the levels of GLUT1 and LDHA was analyzed by western blotting. (**E**) trametinib (50 nM) for 48 h in glioma cells on expression of *GLUT1* and *LDHA* was analyzed by qRT-PCR. Data are presented as means +SD (*n* = 3); *p < 0.05; **p < 0.01, vs control. ^#^ *p* < 0.05; ^##^ *p* < 0.01, vs Tra + vector group. ^&^ *p* < 0.05; ^&&^ *p* < 0.01, vs Tra + PKM2+sh-NC group.

Numerous studies showed that phosphorylated PKM2 can be transferred into the nucleus to regulate the transcription level of c-myc (Yang et al., 2012a). C-myc has also been shown to increase glycolysis by directly binding to GLUT1 and LDHA promoter regions for transcriptional regulation (Dang et al., 2009). Therefore, we wanted to explore whether trametinib can regulate the transcription level of c-myc by regulating PKM2—thus regulating GLUT1 and LDHA expression. Therefore, we further observed the changes of c-myc expression in U87 and U251 treated with trametinib. These discoveries showed that trametinib could significantly inhibit c-myc expression in U87 and U251 cell lines (Figure 3C and Supplementary Figure S3B). Further study found that overexpression of *PKM2* could significantly increase the expression of c-myc with trametinib treatment. To further explore the role of c-myc in killing glioma cells and inhibiting the glycolysis level by trametinib treatment, we transfected sh-myc into the stable strains of two cell lines with *PKM2* overexpression. Next, we examined the migration (Figure 5A), invasion ability (Figure 5B), and glycolysis level (Figure 5C) of these cells. We found that the migration, invasion ability, and glycolysis level of cells were reversed by sh-myc. Expression of *GLUT1* and *LDHA* was also reversed by sh-myc at the transcription level (Figure 5E) and protein level (Figure 5D). These results indicate that trametinib can regulate the glycolysis level, migration, and invasion ability of glioma cells through the PKM2/c-myc axis.

### Trametinib Inhibits the Growth of Glioma Tumors *in vivo*

**FIGURE 6:**
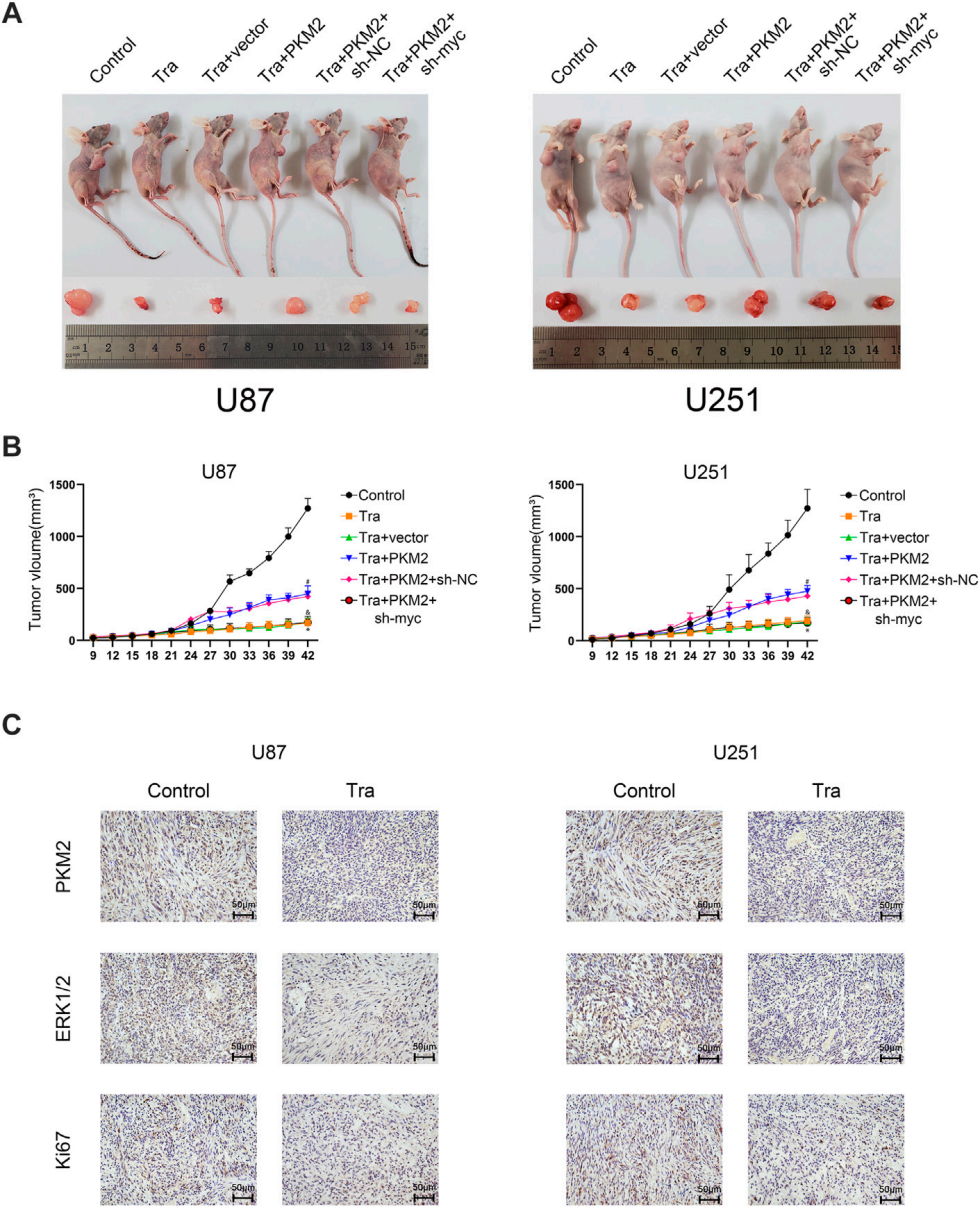
Trametinib inhibits the growth of glioma tumors *in vivo*. (**A**) The size of subcutaneous xenograft tumor in each group of nude mice. (**B**) Changes in the size of subcutaneous transplanted tumors in each group on day 42. One-way analysis of variance was used for statistical analysis. (**C**) trametinib inhibits the expression of Ki67, ERK, and PKM2 *in vivo*. Scale bars: 50 µm Data are presented as means +SD (*n* = 3); *p < 0.05; **p < 0.01, vs control. ^#^ *p* < 0.05; ^##^ *p* < 0.01, vs Tra + vector group. ^&^ *p* < 0.05; ^&&^ *p* < 0.01 vs Tra + PKM2+sh-NC group.

To research the effect of trametinib on tumor growth *in vivo*, we established a transplanted tumor in an athymic nude mice model. U87 and U251 cell lines were planted in nude mice, and each cell line was divided into Control, Tra, Tra + vector, Tra + PKM2+sh-vector, and Tra + PKM2+sh-myc groups. In the first 2 weeks, there was no significant difference between each group. However, the results showed that the tumor size of Tra group was significantly smaller than that of control group. Compared with the Tra group, the tumor size of Tra + vector group had no obvious difference. The Tra + PKM2 group implanted with the *PKM2* overexpression stable strain could partially resist the therapeutic effect of trametinib. Compared with the Tra + PKM2+sh-vector group, the Tra + PKM2+sh-myc group could reduce the antagonistic effect of *PKM2* overexpression on trametinib (Figures 6A,B). The results of immunohistochemistry showed that trametinib could significantly inhibit the levels of Ki67, PKM2, and ERK in glioma cells (Figure 6C). These results indicate that the overexpression of *PKM2* can alleviate the therapeutic effect of trametinib on tumors, while the knock-down of c-myc gene can restore the therapeutic effect of trametinib. In summary, these results illustrate that trametinib can inhibit the size of the glioblastoma xenograft and expression of *Ki67*, *ERK* and *PKM2 in vivo—*which achieve the purpose of treating glioma through the PKM2/c-myc axis.

## Discussion

Trametinib has been evaluated for its anti-cancer effect on metastatic melanoma by inhibiting MEK1 and MEK2 (Lian et al., 2019). Furthermore, trametinib has been approved by the FDA as a monotherapy for the BRAF V600 mutation in unresectable or metastatic melanomas (Lugowska et al., 2015). However, few studies have been published on its basic mechanism in glioma cells. For gliomas, it is difficult to cure with traditional treatment methods. At present, clinical drugs are rarely used to treat glioma making it particularly important to find an effective drug. Nevertheless, trametinib has shown certain therapeutic effects on recurrent/progressive PLGGS (Manoharan et al., 2020). This study aimed to provide new insight for developing clinical drugs to treat glioma. Most previous studies focused on the *in vitro* trials of trametinib in other tumors, and ignored glycolysis—which plays a key role in tumorigenesis. Therefore, the mechanism by which trametinib kills gliomas and its effect on glycolysis level of glioma cells remains to be elucidated.

In our research, we investigated the effect of trametinib on the biological behavior and glycolysis level of glioma cells and its mechanism. Aerobic glycolysis plays a key role in the proliferation and occurrence of cancer cells. By reviewing the existing literature, we found U87 and U251 cell lines are both high-grade malignant glioma cell lines. They have stronger migration and invasion capabilities. Therefore, we chose U87 and U251 cells for further experimentation. Next, we measured the time-dependent change in glycolysis level of glioma cells with trametinib treatment. As treatment time progressed, the glycolysis levels of the two cell lines significantly decreased to varying extents. Our research proved that trametinib inhibits the proliferation, migration, and invasion in glioma cells. Trametinib also induced apoptosis and inhibited intracellular glycolysis-related proteins, including PKM2, GLUT1, and LDHA. Moreover, the inhibitory effects of trametinib treatment on migration, invasion, and glycolysis in glioma cells, together with the induction of apoptosis, were abolished by *PKM2* overexpression—indicating that trametinib treatment efficacy is dependent on PKM2 levels. Furthermore, we observed that the expression of the *c-myc* gene decreased with trametinib treatment, while the overexpression of *PKM2* abolished the expression of *c-myc*. Previous research showed that inhibition of the *c-myc* gene resulted in a decrease in proliferation, migration, invasion ability, and glycolysis level in glioma cells (Zhang et al., 2020). Therefore, we conclude that trametinib suppresses the expression of *PKM2*, which decreases of the expression of *c-myc*, as well as cell proliferation, migration, invasion, and glycolysis level in glioma. Our study showed that the influence of trametinib treatment on glioma is time-dependent. However, we found that the proliferation ability of the two glioma cell lines had different degrees of resistance 24 h after trametinib treatment (Figure 1A). Meanwhile, we observed that PKM2, c-myc, GLUT1, and LDHA expression of the two glioma cell lines reverted at 24 h. This shows that glioma cells may have potential resistant mechanisms to trametinib treatment—requiring further investigation. Studies have shown that trametinib can induce autophagy flux in tumor cells to produce a protective mechanism when tumor cells are inhibited by RAF-MEK-ERK (Kinsey et al., 2019). Therefore, glioma cells could have a similar protective effect (induction of autophagy) against the parameters investigated in this study. However, this view still needs to be verified by future experiments.

Multiple evidence shows that the abnormal expression of the PKM2/c-myc pathway is related to oncogenesis and a change of glycolysis level (Chen et al., 2018). PKM2 is a subtype of pyruvate kinase, which has either a tetramer or dimer form. For instance, the dimeric PKM2 can be transported to the nucleus to function. The transition between the tetramer and dimer form plays an essential role in invasion, metabolism, and cell proliferation (Zhang et al., 2019). Our study showed that the expression of PKM2 increased in the cytoplasm and decreased in the nucleus after trametinib treatment in gliomas. To explore the effect of trametinib on PKM2 in glioma cells, we overexpressed *PKM2* in U87 and U251 cells. The results showed that *PKM2* overexpression could abolish the decrease of the glucose metabolism-related proteins GLUT1 and LDHA, and decrease the intracellular glucose metabolism level, which were influenced by trametinib. These results showed that trametinib inhibited the intracellular glucose metabolism level in glioma cells through PKM2.

The oncogene of the MYC family is the main driving factor of human tumorigenesis, which is mostly overexpressed in tumor tissues compared to normal tissues, and correlates with poor prognosis (Baluapuri et al., 2020). The *MYC* gene has been reported to be highly expressed in gliomas, which can promote cell growth and increase cell sugar fermentation. Numerous reports have shown that *MYC* expression can be reduced by targeting specific metabolic pathways (Collins, 1995; Hsieh et al., 2015). After phosphorylation, PKM2 can be transferred into the nucleus which then function to increase the expression of c-myc. Meanwhile, c-myc can increase GLUT1, PKM2, and LDHA expression (Yang and Lu, 2013). To study whether trametinib activates the expression of c-myc through PKM2, we determined the interaction between PKM2 and c-myc. The fact that trametinib can suppress the expression of c-myc at both the protein and mRNA levels indicates a regulation effect at the transcription level, whereas overexpression of *PKM2* can eliminate the inhibition of trametinib treatment on c-myc. When we knocked out *c-myc* by *PKM2* overexpression, we discovered c-myc suppression with trametinib treatment could restore the increased migration, invasion, and glycolysis level caused by overexpression *PKM2* overexpression. These results indicated that there is a definite interaction between PKM2 and c-myc.

This is the first time that trametinib have shown to inhibit PKM2 and c-myc in glioma cells, but the potential mechanism of the interaction between PKM2 and c-myc is unknown. Previous studies have shown that phosphorylated PKM2 (Ser37) is translocated from the cytoplasm to the nucleus, that the activated PKM2 can combine with histone H3 to phosphorylate H3T11, and then dissociate histone H3K9 from histone HDAC to acetylate H3K9, thus regulating the transcription level of c-myc (Yang et al., 2012b). Other studies have confirmed that phosphorylated PKM2 (Ser37) can also combine with phosphorylated β-catenin c-Src-Y333 to regulate the transcription level of c-myc (Dang et al., 2009). Therefore, trametinib may regulate the interaction between PKM2 and c-myc in two ways: one is to influence the binding of PKM2 to histone H3, and the other is to influence the binding of PKM2 to phosphorylated β-catenin to regulate c-myc. We also used U87 and U251 transplanted tumor models to determine the effect of trametinib on glioma cell growth *in vivo*. We observed that trametinib treatment significantly reduced the growth of glioma cells, also shown *in vitro*. Simultaneously, we also observed that *PKM2* overexpression in glioma cells resisted the effects of trametinib treatment *in vivo*, while glioma cells co-transfected with *PKM2* overexpression and *c-myc* knockout could restore the initial therapeutic effects of trametinib treatment hindered by *PKM2* overexpression. These findings are also consistent with the results observed *in vitro*.

In conclusion, this study reveals a novel role of trametinib in inhibiting glioma cells. Trametinib inhibited the glycolysis level of glioma cells through the PKM2/c-myc pathway, and thus inhibited glioma cells to proliferate, migrate, and invade. At the molecular level, it was shown that the glycolysis level of trametinib on glioma cells is related to the PKM2/c-myc pathway. These results not only indicate the possible mechanism of trametinib in anti-tumor activity, but also reveal the therapeutic potential of trametinib on gliomas, which offers new insight for clinical drug development of gliomas.

## Data Availability

The datasets presented in this study can be found in online repositories. The names of the repository/repositories and accession number(s) can be found below: https://pubchem.ncbi.nlm.nih.gov/, 11707110; http://www.wwpdb.org/,3GR4.
