# Supplementary material for: Trametinib Inhibits the Growth and Aerobic Glycolysis of Glioma Cells by Targeting the PKM2/c-Myc Axis
Source: Front Pharmacol. 2021 Oct 21;12:760055. doi: 10.3389/fphar.2021.760055 (PMC8566436; doi:10.3389/fphar.2021.760055)
Supplement: Supplementary file 1 [file DataSheet1.docx]

**Supplementary Materials**

**1.Figure S1**

**Supplementary Figure 1.** KEGG enrichment analysis of trametinib target **
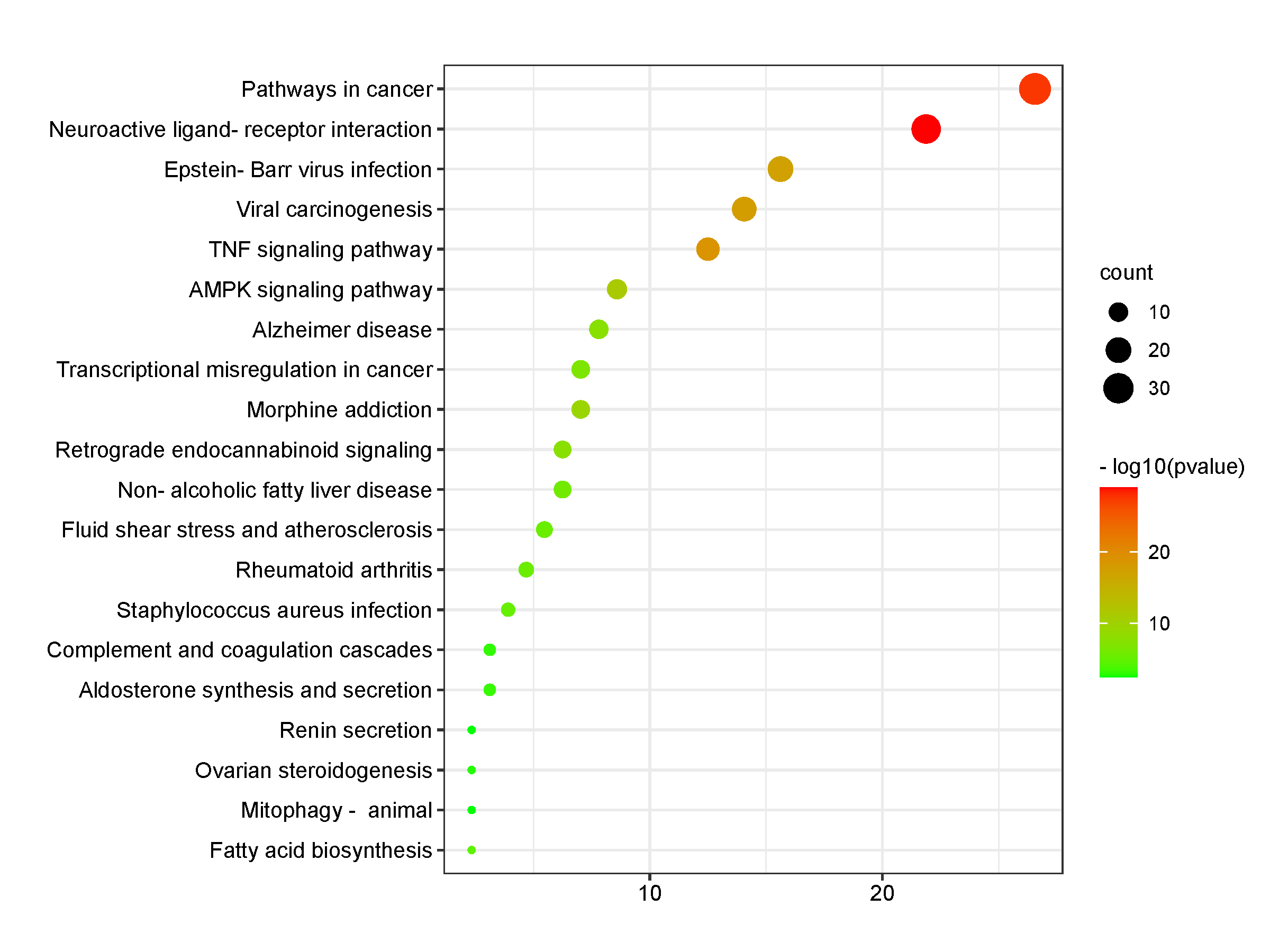
**


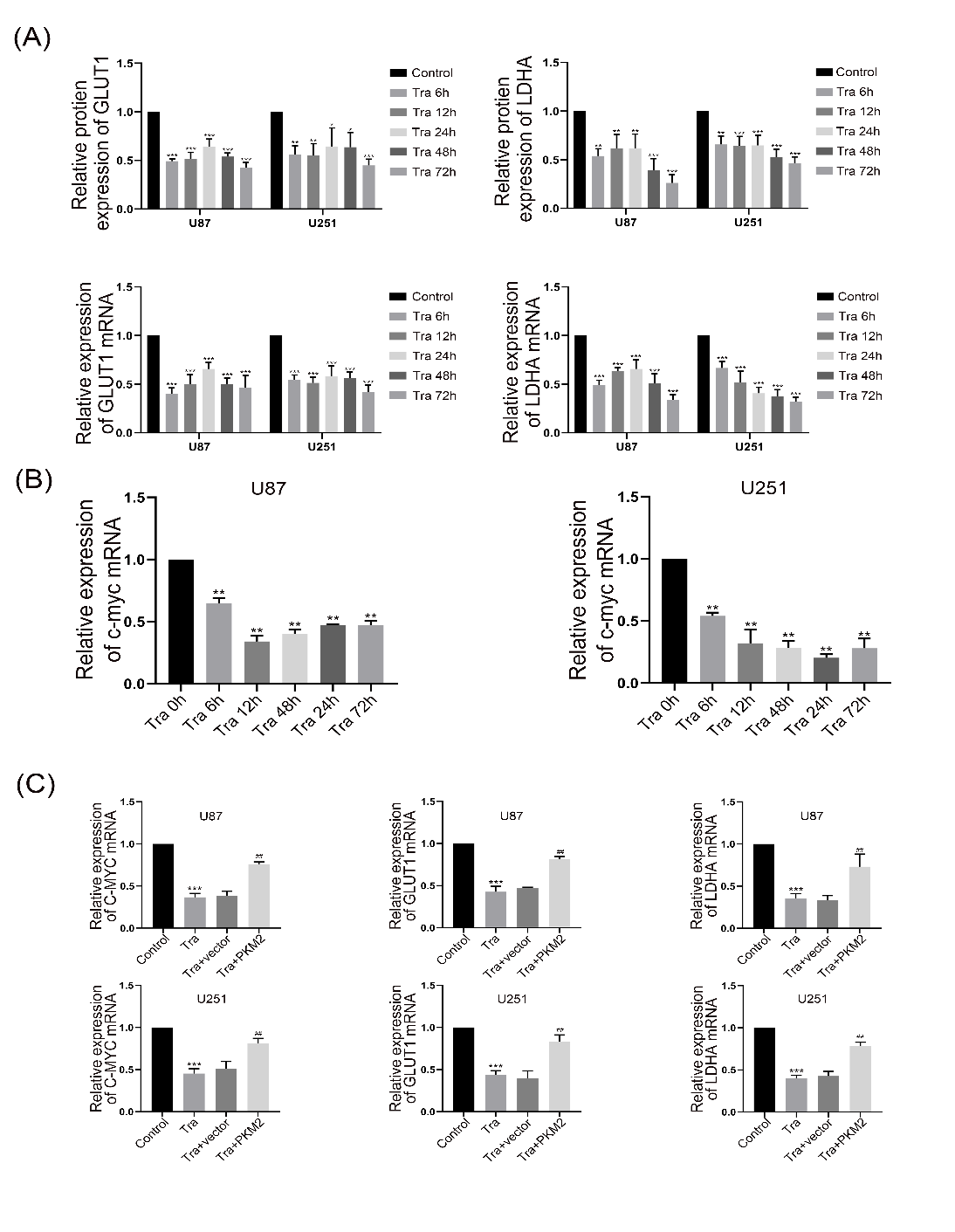
**
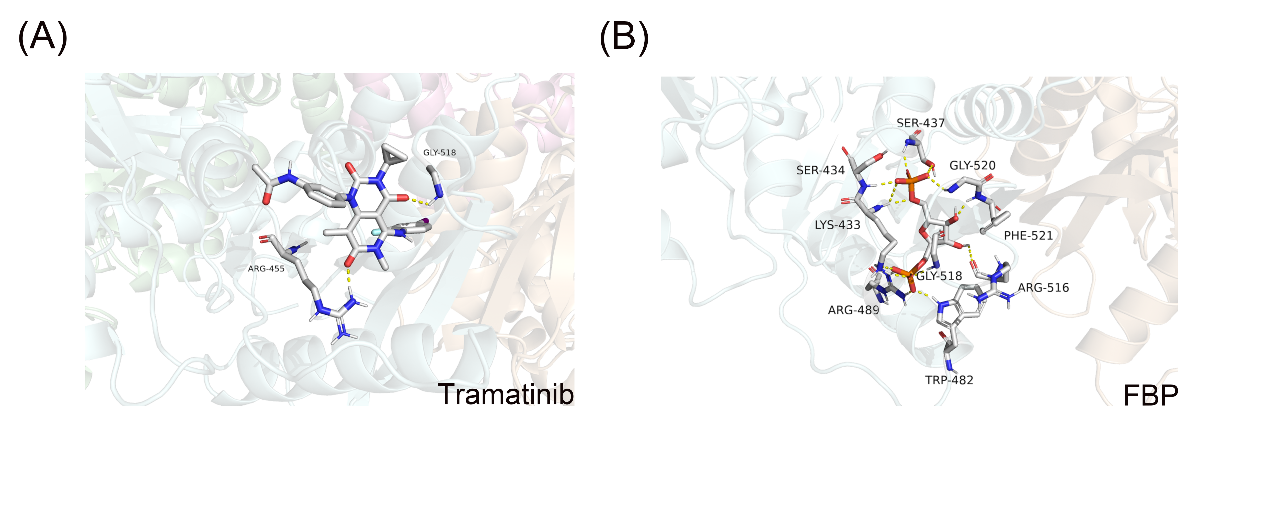
2.Figure S2**

**Supplementary Figure 2.** The binding pocket of ADP in the PKM2 tetramer

**3.Figure S3**

**Supplementary Figure 3.** (A) The mRNA and protein expression of GLUT1 and LDHA in were measured by RT-qPCR after treatment with 50 nM trametinib for 0, 6, 12, 24, 48, and 72 h (B) The mRNA levels of c-myc in U87 and U251 cells were measured by RT-qPCR after treatment with 50nM trametinib for 0h, 6h, 12h, 24h 48h, 72h.(C) c-myc mRNA level was detected in U87 and U251 cells transfected with vector or PKM2 by qRT-PCR assay. Each value represents the mean±SD (n = 3), *P < 0.05, **P < 0.01, compared with control group, #P < 0.05, ##P < 0.01 versus Tra+vector group.
